# Retraction: Isolation and Characterization of a “phiKMV-Like” Bacteriophage and Its Therapeutic Effect on Mink Hemorrhagic Pneumonia

**DOI:** 10.1371/journal.pone.0263042

**Published:** 2022-01-20

**Authors:** The PLOS ONE Editors

During the peer review process and following the publication of this article [1], concerns were raised regarding the welfare of the animals used in the study due to the methods used for anesthesia and euthanasia. In consultation with a member of *PLOS ONE*’s Animal Research Advisory Group, the following concerns were noted:

- Chloral hydrate was used as an anesthetic agent despite being considered to be unreliable for anesthesia.
- Chloral hydrate was administered by intraperitoneal injection despite being described as a peritoneal irritant in the methods section.
- Carbon dioxide use has been associated with pulmonary hemorrhage in other species, and is not considered to be an acceptable method of euthanasia for carnivores used for scientific purposes [2].
- The humane endpoints described in the article may not have been sufficient to prevent avoidable suffering.

The corresponding author stated that they followed local regulations regarding the use of chloral hydrate for anesthesia. Additionally, the corresponding author mentioned that symptoms of abdominal discomfort were not identified in animals following intraperitoneal administration of chloral hydrate, however, they did not consider the potential risk of discomfort and pain when the study was performed.

The corresponding author commented that experiments were performed on a fur farm and followed local guidelines for euthanasia of mink on fur farms [3,4]. The authors noted that while lung hemorrhage was observed in [1], they do not believe it was due to carbon dioxide exposure, and they provide the following reference in in support of this [5].

The member of *PLOS ONE*’s Animal Research Advisory Group advised that the authors’ comments did not resolve the concerns. They noted that the use of chloral hydrate was not in line with ethical standards, and that other anesthetics were available at the time that were less aversive and more effective. Additionally, they stated that chloral hydrate was likely to have caused peritoneal inflammation and subsequent pain during the 12 days following anesthesia.

The advisor also noted that earlier indicators of distress such as abnormal lung sounds, abdominal sensitivity, or reduction in food/water intake, body temperature and body weight should have been considered as humane endpoints. The advisor commented that appropriate veterinary advice should always be sought when deaths are expected or occur in an experiment.

Research published in *PLOS ONE* must have been conducted to the highest ethical standards. Therefore, in light of the above ethical concerns, the *PLOS ONE* Editors retract this article. The editors regret that these concerns were not addressed at the time of the original review process.

ZC, JZ, YDN, YM, FC and YX did not agree with the retraction and stand by the published results. The corresponding author noted that LJ is deceased and all other living authors disagree with the retraction decision.
